# Association of visceral fat metabolism score with risk of rheumatoid arthritis in US adults

**DOI:** 10.3389/fnut.2025.1544624

**Published:** 2025-03-20

**Authors:** Yonghui Li, Yujuan Zhu, Xinwen Tang, Zhiwen Guo, Jian Li, Shuchi Lv, Meng Liu, Yanjie Yu, Changbin Lei

**Affiliations:** ^1^Department of Orthopedics, Affiliated Hospital of Xiangnan University (Clinical College), Chenzhou, China; ^2^Department of Clinical Pharmacy, Affiliated Hospital of Xiangnan University (Clinical College), Chenzhou, China; ^3^Department of Orthopedics, Dongguan Humen Hospital, Dongguan, China

**Keywords:** METS-VF, rheumatoid arthritis, NHANES, obesity, visceral fat metabolism

## Abstract

**Background:**

The Visceral Adiposity Index (METS-VF) has emerged as a novel obesity assessment metric. However, research exploring the relationship between METS-VF and rheumatoid arthritis (RA) remains limited. The objective of this investigation was to examine the correlation between the prevalence of RA and METS-VF.

**Methods:**

The NHANES data collected between 2011 and 2018 were used in this investigation. To determine the association between METS-VF and the prevalence of RA, logistic regression analysis was used. Sensitivity and subgroup analysis were done to test how reliable the results were. Finally, the predictive power of BMI, waist circumference, and METS-VF for RA was compared using ROC curve analysis.

**Results:**

This research had 8,626 individuals in total. The findings showed that compared to those without RA, persons with the condition had noticeably greater METS-VF levels. METS-VF and the prevalence of RA were significantly positively correlated, according to a logistic regression analysis (OR = 1.50, 95% CI = 1.12–2.00). The results of the sensitivity and subgroup analyses agreed with the primary conclusions. ROC analysis indicated that METS-VF possessed a superior ability to predict RA compared to BMI and waist circumference.

**Conclusion:**

This study indicates that elevated METS-VF levels are positively correlated with an increased risk of RA in the US population. Monitoring this metric might aid in the early detection of high-risk patients.

## 1 Background

A common chronic autoimmune disease, rheumatoid arthritis (RA) is characterized by the immune system’s aberrant damage to the joints, hence causing continuous inflammation, discomfort, and joint degradation (1, 2). About 1% of persons worldwide are thought to have RA, and female compared to male prevalence of this condition is clearly higher (3, 4). However, RA not only causes joint pain, stiffness, and swelling, but it can also lead to severe joint deformities and loss of function, thereby limiting patients’ ability to perform daily activities (5, 6). In the long term, RA has been associated with various systemic complications, including cardiovascular diseases, which significantly affect patients’ quality of life (7, 8). Additionally, the decline in work capacity and the increase in medical costs due to RA impose substantial economic burdens on society and healthcare systems (9). Hence, it is essential to thoroughly examine the etiology of RA and its related risk factors to promptly identify groups at high risk and devise more efficient measures for prevention and treatment.

Obesity has become a widespread global health issue, currently affecting more than 2 billion people (10). Obesity is often associated with numerous adverse health effects, and the link between obesity and RA has been attracting increasing research attention. The Body Mass Index (BMI) is often used as a measure to evaluate obesity. However, much research has shown that BMI fails to effectively differentiate between the distribution of muscle and fat, which frequently results in the “obesity paradox” (11). Other anthropometric measurements, such as waist circumference (WC) and waist-to-height ratio (WHtR), are similarly restricted in their capacity to accurately differentiate between visceral fat and subcutaneous fat in the abdominal region (12). The accumulation of visceral adipose tissue (VAT) and insulin resistance (IR) are strongly correlated, as evidenced by recent research (13, 14). Therefore, the development of new alternative indicators to assess obesity more accurately is particularly important. METS-VF combines the Metabolic Score for Insulin Resistance (METS-IR), WHtR, age, and sex to assess visceral fat and metabolic condition together (15). Prior investigations have shown a strong correlation between METS-VF and many chronic illnesses, and its validity has been confirmed in diverse systemic disorders. A cross-sectional research conducted on a Chinese population revealed that elevated METS-VF levels are directly linked to a greater likelihood of developing chronic kidney disease (CKD) and may be used as a valuable clinical marker for detecting CKD (16). Additionally, a separate research revealed a significant positive association between METS-VF and the chance of developing diabetes, indicating that it might be a more effective risk indicator for forecasting the future prevalence of diabetes (12). Subsequent research has shown that METS-VF offers a superior evaluation in comparison to other central obesity indicators (17). Nevertheless, the relationship between METS-VF and RA remains incompletely understood. Previous studies have shown a link between visceral adiposity and various health conditions, including RA. A recent study highlighted the role of visceral fat in RA-related complications, demonstrating that the Visceral Adiposity Index (VAI) predicts trabecular bone loss in female patients with early RA. The aim of this study is to examine the association between METS-VF and RA using data from NHANES. This study aims to provide innovative viewpoints and methodologies for promptly identifying individuals who are more susceptible to acquiring RA.

## 2 Materials and methods

### 2.1 Study design and population

The National Center for Health Statistics (NCHS) of the CDC conducts the National Health and Nutrition Examination Survey (NHANES) to assess health and nutrition of the US population. All individuals gave written informed permission after the NCHS Ethics Review Board approved the study. Four NHANES cycles from 2011 to 2018 were analyzed. The participants were chosen according to specific inclusion criteria: (1) Individual must be at least 20 years of age. (2) Have complete information on RA. (3) Have detailed information relating to METS-VF.

### 2.2 Definition of METS-VF

Health technicians with the necessary qualifications at the Mobile Examination Center (MEC) evaluated the participants’ BMI, waist circumference (WC), and body height (BH). The Cobas 6000 chemical analyzer was used to quantify triglycerides (TG) and high-density lipoprotein cholesterol (HDL-C), whereas the Roche/Hitachi Cobas C chemistry analyzer C311 was used to quantify fasting blood glucose (FBG). Provided below are the mathematical equations for computation:


$$W\,H\,t\,R=\frac{W\,C\,\left(c\,m\right)}{B\,H\,\left(c\,m\right)}$$

$$M\,E\,T\,S-I\,R=\frac{\left(L\,n\,\left(\left(2\times F\,B\,G\right)+T\,G\right)\times B\,M\,I\right)}{\left(L\,n\,\left(H\,D\,L-C\right)\right)}$$

$$M\,E\,T\,S-V\,F=4.466+0.01\times \left[{\left(L\,n\,\left(M\,E\,T\,S-I\,R\right)\right)}^{3}\right]+3.239$$

$$\times \left[{\left(L\,n\,\left(W\,H\,t\,R\right)\right)}^{3}\right]+0.319\times S\,e\,x+0.594\times \left(L\,n\,\left(A\,g\,e\right)\right)$$

In this study, the sex variable was coded as 0 for male participants and 1 for female participants. WC and BH were measured in centimeters, while FBG, TG, and HDL-C were expressed in mg/dL.

### 2.3 Definition of RA

RA patients were identified based on the following two questions: “Has a doctor ever told you that you have arthritis?” If the participant answered affirmatively, they were then asked, “What type of arthritis do you have?” If the participant responded with “RA,” they were classified as having RA. Previous studies have validated the feasibility of self-reported RA (18, 19).

### 2.4 Covariates

This study included demographic characteristics, lifestyle factors, and chronic diseases as covariates for model adjustment. Demographic characteristics included age, sex, race, and education level. Lifestyle factors included smoking, drinking, and physical activity. To be classified as smokers, persons were required to have consumed a minimum of 100 cigarettes over their lives. Drinkers were classified as those who had ingested any number of alcoholic drinks over 12 during the previous year. Physical activity was calculated based on metabolic equivalents (METs) from the physical activity questionnaire; individuals with METs < 600 were classified as inactive. The formula used was: MET (minutes/week) = MET × weekly frequency × duration per session. Chronic diseases were determined based on self-report and included coronary heart disease, chronic kidney disease, hypertension, and diabetes.

### 2.5 Statistical analysis

This study conducted a cross-sectional analysis using information from four NHANES cycles, which span the years 2011 through 2018. The clinical baseline features of the study population were categorized according to the condition of their RA. A study using logistic regression analysis was carried out to investigate the association between METS-VF and the prevalence of RA. To examine the correlation between varying levels of METS-VF and the prevalence of RA, the METS-VF variable was divided into four quartiles. The Q1 (<6.44), Q2 (6.44–6.95), Q3 (6.95–7.35), and Q4 (>7.35) quartiles were defined as follows. The variables in Model 1 were unchanged. Age, sex, and ethnicity were included as variables in Model 2. All relevant factors were included in Model 3, including age, sex, race, education level, physical activity, smoking, drinking, and conditions including diabetes, hypertension, and coronary artery disease (CAD). An investigation of restricted cubic splines (RCS) was conducted to assess potential dose-response relationships between RA and METS-VF. Sensitivity analysis was used to exclude extreme METS-VF values in order to increase the results’ robustness. Subgroup analysis was also carried out to assess potential METS-VF influencing factors on RA in different populations. To assess the predictive qualities of BMI, WC, and METS-VF in connection to RA, ROC curve analysis was done. R software (version 4.2.3) was used to conduct the studies, and a significance criterion of *P* < 0.05 was specified.

## 3 Results

### 3.1 Baseline characteristics of participants

**FIGURE 1:**
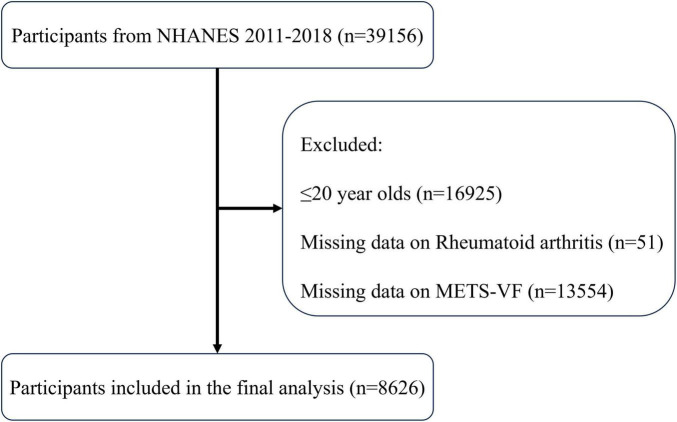
Flowchart of the sample selection from NHANES.

The protocol for selecting participants is shown in Figure 1, which led to the incorporation of 8,626 individuals, consisting of 8,187 people without RA and 439 patients with RA. An overview of the individuals’ baseline characteristics is provided in Table 1. In comparison to the non-RA group, RA patients exhibited higher age, a greater likelihood of a smoking history, and a more sedentary lifestyle. Furthermore, they had a higher susceptibility to diabetes, coronary heart disease, and chronic renal disease. Furthermore, patients with RA showed notably elevated levels of METS-VF compared to those without RA, indicating a potential correlation between METS-VF and the prevalence of RA.

**TABLE 1:** Baseline characteristics of the study population.

Characteristic	Level	Overall	Non-RA	RA	*P*-value
*n*		8626	8187	439	
Age (%)	<50	4388 (50.9)	4292 (52.4)	96 (21.9)	<0.001
	>50	4238 (49.1)	3895 (47.6)	343 (78.1)	
Sex (%)	Female	4424 (51.3)	4186 (51.1)	238 (54.2)	0.226
	Male	4202 (48.7)	4001 (48.9)	201 (45.8)	
Race (%)	Mexican American	1195 (13.9)	1132 (13.8)	63 (14.4)	<0.001
	Non-Hispanic Black	1782 (20.7)	1643 (20.1)	139 (31.7)	
	Non-Hispanic White	3261 (37.8)	3110 (38.0)	151 (34.4)	
	Others	2388 (27.7)	2302 (28.1)	86 (19.6)	
Education level (%)	Under high school	1899 (22.0)	1769 (21.6)	130 (29.6)	<0.001
	High school	1884 (21.8)	1779 (21.7)	105 (23.9)	
	Above high school	4840 (56.1)	4637 (56.6)	203 (46.2)	
	No record	3(0.0)	2 (0.0)	1 (0.2)	
Smoke (%)	No	4892 (56.7)	4688 (57.3)	204 (46.5)	<0.001
	Yes	3726 (43.2)	3493 (42.7)	233 (53.1)	
	No record	8 (0.1)	6 (0.1)	2 (0.5)	
Drinking (%)	No	1869 (23.2)	1751 (22.9)	118 (28.3)	0.039
	Yes	6175 (76.7)	5876 (77.0)	299 (71.7)	
	No record	3 (0.0)	3 (0.0)	0 (0.0)	
Activity status (%)	Active	4611 (53.5)	4407 (53.8)	204 (46.5)	0.003
	Inactive	4015 (46.5)	3780 (46.2)	235 (53.5)	
Diabetes (%)	No	7177 (83.2)	6873 (84.0)	304 (69.2)	<0.001
	Yes	1205 (14.0)	1088 (13.3)	117 (26.7)	
	No record	244 (2.8)	226 (2.8)	18 (4.1)	
CAD (%)	No	8015 (92.9)	7659 (93.6)	356 (81.1)	<0.001
	Yes	611 (7.1)	528 (6.4)	83 (18.9)	
BMI [mean (SD)] (kg/m^2^)		29.30 (6.96)	29.21 (6.93)	31.10 (7.40)	<0.001
HDL [mean (SD)] (mg/dL)		53.89 (15.99)	53.89 (16.03)	53.89 (15.28)	0.999
TC [mean (SD)] (mgl/dL)		190.05 (41.63)	190.20 (41.68)	187.28 (40.55)	0.152
GLU [mean (SD)] (mg/dL)		110.58 (36.02)	110.26 (35.89)	116.72 (37.90)	<0.001
TG [mean (SD)] (mg/dL)		119.60 (107.38)	119.59 (108.75)	119.77 (77.46)	0.972
WC [mean (SD)] (cm)		99.89 (16.57)	99.61 (16.51)	105.23 (16.75)	<0.001
BH [mean (SD)] (cm)		166.95 (9.98)	167.04 (9.95)	165.23 (10.31)	<0.001
METS_VF [mean (SD)]		6.82 (0.73)	6.80 (0.73)	7.18 (0.56)	<0.001

Mean (SD) for continuous variables, % for categorical variables. ALT, Alanine Aminotransferase; AST, Aspartate Aminotransferase; BH, body height; BMI, Body Mass Index; BUN, Blood Urea Nitrogen; CAD, Coronary Artery Disease; GLU, glucose; METS-IR, metabolic score for insulin resistance; METS-VF, metabolic score for visceral fat; RBC, red blood cell count; SUA, serum uric acid; TC, total cholesterol; TG, triglyceride; WBC, white blood cell count; WC, Waist Circumference; WHtR, waist-to-height ratio.

### 3.2 Association between METS-VF and RA prevalence

**FIGURE 2:**
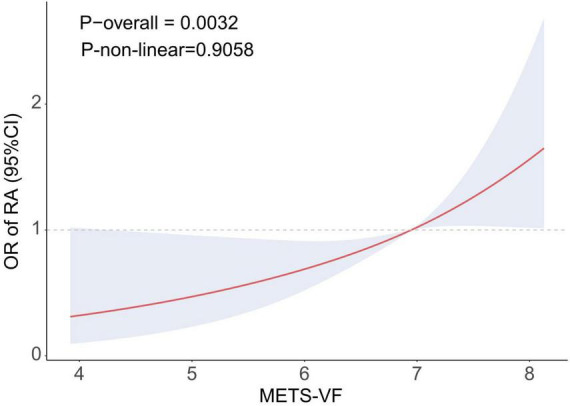
RCS curve illustrating the association between METS-VF and RA. METS-VF, metabolic score for visceral fat; OR, odds ratio; RA, rheumatoid arthritis; Adjusted for age, sex, race, education, smoking, drinking, activity status, CAD, diabetes, and hypertension.

The results of the logistic regression investigation on the correlation between METS-VF and the prevalence of RA are presented in Table 2. The results indicated a robust positive correlation (OR: 2.30, 95% CI: 1.81–2.92) between the prevalence of RA and METS-VF. The high connection remained statistically significant even after taking into consideration a number of variables (OR: 1.50, 95% CI: 1.12–2.00). Following quartile classification, the highest quartile (OR: 2.28, 95% CI: 1.17–4.42) was shown to have a greater connection with the prevalence of RA than the lowest quartile (RA prevalence). The RCS analysis findings are shown in Figure 2, which shows a strong correlation between the prevalence of RA and an increase in METS-VF. The results point to a long-lasting and favorable correlation between METS-VF and RA prevalence.

**TABLE 2:** The relationship between METS-VF and RA.

		Model 1 OR (95%CI) *P*-value	Model 2 OR (95%CI) *P*-value	Model 3 OR (95%CI) *P*-value
RA	METS-VF	2.30 (1.81, 2.92) < 0.001	1.82 (1.35, 2.45) < 0.001	1.50 (1.12, 2.00) 0.007
	Q1	[Reference]	[Reference]	[Reference]
	Q2	2.50 (1.30, 4.80) 0.007	1.98 (1.04, 3.76) 0.037	1.77 (0.90, 3.50) 0.100
	Q3	2.11 (1.17, 3.80) 0.013	1.51 (0.84, 2.71) 0.200	1.24 (0.69, 2.23) 0.500
	Q4	4.87 (2.76, 8.60) < 0.001	3.10 (1.65, 5.81) < 0.001	2.28 (1.17, 4.42) 0.016
	P for trend	<0.001	<0.001	0.022

BMI, Body Mass Index; CI, Confidence Interval; METS-VF, metabolic score for visceral fat; OR, Odds Ratio; WC, Waist Circumference; Model 1: No covariates adjusted; Model 2: Adjusted for Age, Sex, and Race; Model 3: Adjusted for Age, Sex, Race, Educational level, Smoke, Drinking, Activity status, CAD, Diabetes, Hypertension.

### 3.3 Subgroup and sensitivity analyses

Subgroup and sensitivity analyses were implemented to verify the reliability of the research findings. The subgroup analysis in Table 3 showed that the positive correlation between METS-VF and the prevalence of RA remained statistically significant among individuals who were under 50 years old, male, and did not have any chronic conditions. The interaction tests revealed no statistically significant interactions. A sensitivity analysis was performed on the remaining 8,540 individuals by eliminating high METS-VF results (±3 SD). The results indicated that the strong correlation between METS-VF and the prevalence of RA persisted (Supplementary Table 1), providing further affirmation for the primary conclusions of this investigation.

**TABLE 3:** Subgroup analysis between METS-VF and RA.

Characteristic	Group	OR (95%CI) *P*-value	P for interaction
Age	<50	2.02 (1.14, 3.58) 0.017	0.073
	>50	1.19 (0.85, 1.68) 0.300	
Sex	Male	1.76 (1.13, 2.73) 0.013	>0.900
	Female	1.29 (0.86, 1.91) 0.200	
Race	Mexican American	2.64 (1.05, 6.63) 0.039	0.300
	Non-Hispanic Black	1.44 (0.95, 2.18) 0.081	
	Non-Hispanic White	1.48 (0.96, 2.27) 0.074	
	Others	1.52 (0.72, 3.22) 0.300	
Hypertension	No	1.60 (1.08, 2.37) 0.019	0.800
	Yes	1.31 (0.76, 2.25) 0.300	
Diabetes	No	1.43 (1.06, 1.92) 0.020	0.400
	Yes	2.36 (0.79, 7.05) 0.120	
CAD	No	1.57 (1.14, 2.16) 0.007	>0.900
	Yes	1.23 (0.54, 2.80) 0.600	

### 3.4 ROC analysis

**FIGURE 3:**
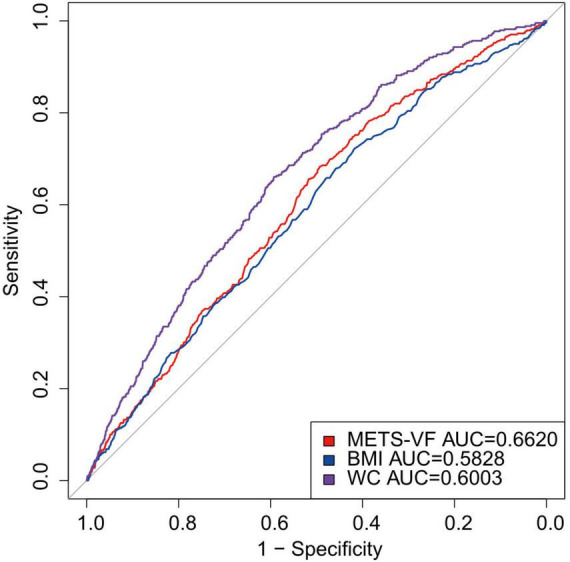
Diagnostic performance of obesity METS-VF, BMI, WC index on RA prevalence. BMI, Body Mass Index; METS-VF, metabolic score for visceral fat; WC, waist circumference.

Figure 3 displays the ROC curves and AUC values pertaining to the prediction of RA. The findings indicated that the Area Under the Curve (AUC) for METS-VF was 0.6620, therefore surpassing the AUC values of BMI (0.5828) and waist circumference (0.6003) by a substantial margin. Therefore, METS-VF demonstrates superiority over conventional obesity markers in forecasting the likelihood of developing prevention of RA. The results imply that METS-VF outperforms conventional obesity markers in forecasting the likelihood of developing RA. This implies that METS-VF has the potential to be used in clinical settings for early detection and prevention of rheumatoid conditions.

## 4 Discussion

This study examined the correlation between the prevalence of RA and METS-VF using data from the NHANES database. In the United States, there is a robust correlation between METS-VF and the prevalence of adult RA, as indicated by the research. According to further ROC analysis, METS-VF was more precise in predicting RA than conventional indicators such as BMI and waist circumference. This suggests that METS-VF may have more practical therapeutic applications.

The results of this research demonstrate a strong positive correlation between METS-VF and the prevalence of RA, indicating that visceral fat and related metabolic abnormalities are important factors in the development of RA. High levels of METS-VF not only indicate increased visceral fat but also suggest the presence of metabolic disturbances. The presence of these abnormalities can trigger the release of pro-inflammatory cytokines such as TNF-α and IL-6. This creates an environment that promotes inflammation and activates the immune system, ultimately resulting in joint inflammation and the progression of RA (20, 21). Moreover, METS-VF is associated with insulin resistance, which enhances pro-inflammatory signaling pathways and exacerbates chronic inflammatory responses (13). The confluence of these metabolic anomalies may explain the increased vulnerability of those with elevated METS-VF to RA. These findings are partially consistent with the existing literature, which has established a clear link between obesity and the onset of RA (22, 23). A study reported that abdominal obesity is associated with an increased risk of RA in young and middle-aged women (24). Similarly, another cross-sectional study indicated a nonlinear positive association between WWI and RA prevalence (23). Furthermore, A Mendelian research revealed that genetically determined BMI doubles the risk of RA (25). Compared to traditional indicators like BMI and waist circumference, METS-VF simultaneously reflects the metabolic activity of visceral fat, making it superior in predicting RA risk. The research revealed that the AUC value of METS-VF was notably greater than that of BMI and waist circumference, therefore confirming its efficacy in inferring the risk of RA. These findings underscore the importance of metabolic health in the prevention and management of RA, suggesting that monitoring and managing METS-VF levels can help identify high-risk individuals early and reduce the risk of RA onset by improving the metabolic function of visceral fat. This provides a new perspective for future intervention strategies.

The positive association between METS-VF and RA may result from the interplay of various complex biological mechanisms. As an active endocrine organ, visceral fat’s metabolic abnormalities can lead to the over-secretion of pro-inflammatory cytokines like TNF-α and IL-6. These cytokines play a critical role in the chronic inflammatory response in RA by activating the immune system and promoting the accumulation of inflammatory cells in the synovium, thereby exacerbating inflammation and tissue damage in RA (21, 26, 27). Secondly, METS-VF is closely related to insulin resistance, which not only disrupts normal glucose metabolism but also activates inflammatory signaling pathways such as NF-κB and JAK-STAT, aggravating systemic inflammatory responses (28–30). These pathways are often found to be highly active in RA patients, further promoting the release of inflammatory cytokines and sustaining the inflammatory response. Additionally, abnormal visceral fat metabolism may lead to increased oxidative stress, which can cause lipid peroxidation of cell membranes and DNA damage, thereby disrupting cellular structure and function (31–33). Oxidative stress can also activate immune cells such as macrophages and neutrophils, enhancing their inflammatory responses and further worsening RA symptoms (34, 35). The interaction of these mechanisms places individuals with high METS-VF levels at greater risk for the onset and progression of RA. A deeper understanding of these biological mechanisms not only elucidates the critical role of METS-VF in the pathogenesis of RA but also provides a new scientific basis and direction for developing preventive and therapeutic strategies for RA in the future.

There are several noteworthy strengths in this study. Strong validity and generalizability of the findings to the larger adult population in the United States are facilitated by the NHANES database’s diverse demographic and big sample size. Furthermore, this research highlighted the exceptional efficacy of METS-VF, a comprehensive metabolic scoring instrument, in forecasting the susceptibility to RA. Nevertheless, this study does have certain limitations. Firstly, it is more difficult to determine precise causal correlations due to the study’s cross-sectional methodology. Although we identified an association between METS-VF and RA prevalence, it cannot be determined whether METS-VF directly causes the onset of RA. Moreover, NHANES data are primarily based on the U.S. population, and whether the findings are applicable to other ethnicities or regions requires further investigation. As this study is focused on the U.S. population using NHANES data, future research should consider validating our findings in Asian populations to assess the generalizability of the results. Longitudinal studies in diverse populations will be essential to further explore the causal relationship between METS-VF and RA.

## 5 Conclusion

This study indicates that elevated METS-VF levels are positively correlated with an increased risk of RA in the US population. Monitoring this metric might aid in the early detection of high-risk patients.

## Data Availability

Publicly available datasets were analyzed in this study. This data can be found here: https://www.cdc.gov/nchs/nhanes/index.htm.
