# Supplementary material for: Association of visceral fat metabolism score with risk of rheumatoid arthritis in US adults
Source: Front Nutr. 2025 Mar 20;12:1544624. doi: 10.3389/fnut.2025.1544624 (PMC11974506; doi:10.3389/fnut.2025.1544624)
Supplement: Supplementary file 1 [file Table_1.docx]

**Supplementary Table 1 Sensitivity analysis between METS-VF and RA.**

|  |  | Model 1  OR (95%CI) P-value | Model 2  OR (95%CI) P-value | Model 3  OR (95%CI) P-value |
| --- | --- | --- | --- | --- |
| RA | METS-VF | 2.37 (1.86, 3.03) <0.001 | 1.88 (1.38, 2.56) <0.001 | 1.55 (1.16, 2.08) 0.004 |
|  | Q1 | [Reference] | [Reference] | [Reference] |
|  | Q2 | 2.52 (1.29, 4.91) 0.007 | 2.00 (1.04, 3.85) 0.039 | 1.80 (0.90, 3.59) 0.094 |
|  | Q3 | 2.13 (1.16, 3.90) 0.015 | 1.53 (0.84, 2.78) 0.200 | 1.26 (0.69, 2.29) 0.400 |
|  | Q4 | 4.91 (2.73, 8.83) <0.001 | 3.13 (1.65, 5.95) <0.001 | 2.32 (1.18, 4.54) 0.015 |
|  | P for trend | <0.001 | <0.001 | 0.019 |

BMI: Body Mass Index; CI: Confidence Interval; METS-VF: Metabolic score for visceral fat; OR: Odds Ratio; WC: Waist Circumference

Model 1: No covariates adjusted; Model 2: Adjusted for Age, Sex, and Race; Model 3: Adjusted for Age, Sex, Race, Educational level, Smoke, Drinking, Activity status, CAD, Diabetes, Hypertension.
